# Circulatory HMGB1 is an early predictive and prognostic biomarker of ARDS and mortality in a swine model of polytrauma

**DOI:** 10.3389/fimmu.2023.1227751

**Published:** 2023-07-14

**Authors:** Matthew D. Young, Tomas S. Cancio, Catherine R. Thorpe, Robert P. Willis, John K. Snook, Bryan S. Jordan, Samandra T. Demons, Jose Salinas, Zhangsheng Yang

**Affiliations:** Organ Support Department, United States (US) Army Institute of Surgical Research, Fort Sam Houston, TX, United States

**Keywords:** acute respiratory distress syndrome, high-mobility group box 1, Toll-like receptor 4, inflammation, mortality, biomarker, polytrauma, smoke inhalation

## Abstract

Acute respiratory distress syndrome (ARDS) is a leading cause of morbidity and mortality in polytrauma patients. Pharmacological treatments of ARDS are lacking, and ARDS patients rely on supportive care. Accurate diagnosis of ARDS is vital for early intervention and improved outcomes but is presently delayed up to days. The use of biomarkers for early identification of ARDS development is a potential solution. Inflammatory mediators high-mobility group box 1 (HMGB1), syndecan-1 (SDC-1), and C3a have been previously proposed as potential biomarkers. For this study, we analyzed these biomarkers in animals undergoing smoke inhalation and 40% total body surface area burns, followed by intensive care for 72 h post-injury (PI) to determine their association with ARDS and mortality. We found that the levels of inflammatory mediators in serum were affected, as well as the degree of HMGB1 and Toll-like receptor 4 (TLR4) signal activation in the lung. The results showed significantly increased HMGB1 expression levels in animals that developed ARDS compared with those that did not. Receiver operating characteristic (ROC) analysis showed that HMGB1 levels at 6 h PI were significantly associated with ARDS development (AUROC=0.77) and mortality (AUROC=0.82). Logistic regression analysis revealed that levels of HMGB1 ≥24.10 ng/ml are associated with a 13-fold higher incidence of ARDS [OR:13.57 (2.76–104.3)], whereas the levels of HMGB1 ≥31.39 ng/ml are associated with a 12-fold increase in mortality [OR: 12.00 (2.36–93.47)]. In addition, we found that mesenchymal stem cell (MSC) therapeutic treatment led to a significant decrease in systemic HMGB1 elevation but failed to block SDC-1 and C3a increases. Immunohistochemistry analyses showed that smoke inhalation and burn injury induced the expression of HMGB1 and TLR4 and stimulated co-localization of HMGB1 and TLR4 in the lung. Interestingly, MSC treatment reduced the presence of HMGB1, TLR4, and the HMGB1-TLR4 co-localization. These results show that serum HMGB1 is a prognostic biomarker for predicting the incidence of ARDS and mortality in swine with smoke inhalation and burn injury. Therapeutically blocking HMGB1 signal activation might be an effective approach for attenuating ARDS development in combat casualties or civilian patients.

## 1 Introduction

Smoke inhalation (SI) injury is a leading causal factor of burn injury-related death, with an estimated incidence of 20% to 35% in the US (1–3). Severe or undiagnosed cases of SI and burn injury can lead to acute respiratory distress syndrome (ARDS), resulting in respiratory failure due to hypoxemia (4, 5). ARDS is a life-threatening condition (5, 6). Data from a military study revealed that one-third of patients with burn and smoke inhalation injury develop ARDS, especially in patients who require mechanical ventilation (7). Clinically, ARDS is defined by acute onset of symptoms and the presence of bilateral radiographic opacities of a non-cardiac origin, not fully explained by pulmonary effusions, collapses, or nodules, and a PaO_2_/FiO_2_ ratio (PFR) of less than 300 (8–10). Approximately 200,000 cases of ARDS are diagnosed in the United States annually (11), but the incidence rate is much higher with 3 million patients experiencing ARDS annually with poor clinical outcomes and a pooled mortality rate of up to 46% (11, 12).

The pathological changes of ARDS have been described as taking place over three overlapping phases: the exudative (acute), proliferative (subacute), and fibrotic (chronic) phases (13, 14). In the acute phase, the pathophysiological hallmark of ARDS is unchecked inflammation-driven diffuse alveolar damage (DAD) and alveolar-capillary barrier dysfunction, leading to bilateral infiltration, interstitial and alveolar edema, capillary congestion, intra-alveolar hemorrhage, and inflammatory cell infiltration (13). Traditionally, ARDS is recognized as a neutrophil-driven disease (15). Recently, however, accruing evidence has demonstrated that innate immune cells—such as macrophages and platelets—also play a critical role in the pathogenesis of ARDS (16, 17). Notably, alveolar macrophages (AMs) respond to inflammatory insults from both the lungs and extra-pulmonary sites by recruiting neutrophils and other macrophages—along with bioactive agents including chemokines, proteases, and eicosanoids—to the injury site, encouraging inflammation in the early phase post-injury (PI) (18, 19). An excess of inflammation and the subsequent proliferation of proinflammatory mediators such as interleukin (IL)-1β, IL-6, IL-8, and TNF-α in the inflamed space can cause damage to tissue and cell death. Both alveolar type 1 (AT1) and type 2 (AT2) epithelial cells are critical, as their dysfunction impairs vectorial sodium (Na^+^) transport via epithelial Na^+^ channels (ENaC) and the Na,K-adenosine triphosphatase (Na,K-ATPase) pump (20). Impaired Na^+^ transport reduces fluid clearance capacity of the lung and is the primary cause of impaired gas exchange (21). Likewise, injured AT1 and AT2 cells decrease the production of pulmonary surfactant, also contributing to reduced alveolar fluid clearance and impaired gas exchange (22, 23).

High molecular group box 1 (HMGB1) has been identified as a critical alarmin that serves as a damage-associated molecular pattern (DAMP), inducing a profound inflammatory response after trauma (24). Following trauma, the DAMPs released by the host have been shown to elicit immune responses like those caused by pathogen-associated molecular patterns (PAMP). By interacting with its various receptors—including toll-like receptors (TLRs), the receptor for advanced glycation end products (RAGE), and complement receptors—HMGB1 triggers multiple innate pathways, including nuclear factor-κB (NF-κB) (25), phosphoinositide 3-kinase (PI3k)/akt kinase (AKT) (26), mitogen−activated protein kinase (MAPK) (27), and Janus kinase/signal transducer and activator of transcription (JAK/STAT) (28). The resulting cascades release a broad range of inflammatory cytokines into a cytokine storm that subsequently trigger the systemic inflammatory response syndrome (SIRS) (24). When excessive, this inflammatory response reciprocally with compensatory anti-inflammatory response syndrome (CARS) can lead to endotheliopathy, immune paresis, tissue/organ damage, multiple-organ failure (MOF), and eventually death (29).

Currently, ARDS patients mainly rely on supportive care, such as mechanical ventilation or fluid-conservative therapy (30, 31). These methodologies carry risks to the patient, as mechanical ventilation itself can also be destructive due to the risk of ventilator-induced lung injury (VILI) (32, 33), whereas improper resuscitation can cause hypervolemia and electrolyte imbalance leading to pulmonary edema, nephrotoxicity, or MOF (34). Despite significant advances, improving survival of ARDS patients remains a major challenge, mainly due to the lack of therapeutic targets and delays to diagnosis of ARDS by current clinical standards (35). Several biomarkers have been proposed for predicting ARDS development, such as IL-6, IL-8, RAGE, angiopoietin-2 (Ang-2), Von Willebrand factor (vWF), and surfactant protein D (SP-D) (35, 36). However, due to the heterogeneity in the pathophysiology of ARDS, a rapid, specific, and reliable biomarker for predicting ARDS development is still not available. Our current work attempts to fill this capability gap.

## 2 Materials and methods

Results from all studies used in this analysis were approved by the Institutional Animal Care and Use Committee (IACUC) of the U.S. Army Institute of Surgical Research. Research was conducted in compliance with the Animal Welfare Act, Animal Welfare regulations, and the principles of the Guide for the Care and Use of Laboratory Animals. IACUC reviewed and approved all research conducted in this study. The facility where this research was conducted is fully accredited by the Association for Assessment and Accreditation of Laboratory Animal Care (AAALAC) International.

### 2.1 Animal preparation

All studies followed an established model previously described by our institution, receiving standard surgical preparation and smoke inhalation, and 40% total body surface area (TBSA) burn injuries (37, 38). Briefly, non-pregnant female Yorkshire swine, weighing 35–50 kg, were premedicated with Telazol (tiletamine/zolazepam 6 mg/kg) and glycopyrrolate (0.01 mg/kg), and anesthetized with isoflurane. Animals were then cleaned, shaved, and intubated with an 8–10-French (Fr) endotracheal tube. After that, a Foley catheter was placed. Under sterile conditions, central lines (8–8.5-Fr catheters, Arrow Int’l, Reading, PA) were placed in bilateral jugular and femoral veins and bilateral femoral arteries using the modified Seldinger technique. An open tracheostomy was performed. Following successful placement of at least one central venous line, total intravenous anesthesia (TIVA) was initiated using midazolam, fentanyl, ketamine, and propofol and isoflurane was weaned. Arterial blood pressure was measured through one of the two femoral lines. Following surgical procedures, the animals were allowed to reach equilibrium before baseline (BL) samples were collected. Following BL, all animals received a 40% TBSA full-thickness burn and smoke inhalation injury according to the established model at our institution (37, 38). In brief, cooled wood smoke was delivered to induce the smoke injury, immediately followed by a 20% TBSA full-thickness burn applied using an open flame (Bunsen burner) on each flank. During the injury phase as well as observation period, medications were titrated to effectiveness as needed and continuously applied throughout the duration of the study. All animals were continuously monitored from the start to completion of the study. Appropriate analgesic control was confirmed by adequate toe pinching across the coronary band with a large hemostat, as well as jaw tone assessment to ensure surgical plane of anesthesia.

### 2.2 Study groups and interventions

Animals were randomly assigned to different study groups according to original experimental plans. In total, the samples of 39 animals were included in this current study. The first study consisted of a group of injury control animals with no treatment (n=9), the second study included animals with placebo (saline n=8) and autologous mesenchymal stem cell (MSC) treatment (MSCs, n=9), and the third study included animals with extra-corporeal life support (ECLS) devices, either the Hemolung (ALung Technologies, Pittsburgh, PA) (n=6) or the Novalung (Xenios, Heilbronn, Germany) (n=7). All animals were placed in the intensive care unit (ICU) and observed for 72 h or until death. The animals received burn resuscitation care, including mechanical ventilation (MV), with a BL mechanical ventilation of 10 ml/kg tidal volume (TV) in all groups. Once ARDS developed, MV was titrated according to the ARDSnet protocol to maintain normocarbia (39). For MSC treatment, MSCs were collected from the iliac crest and femur of the swine. Collected bone marrow was concentrated using a bedside cell concentrator (Magellan, Arteriocyte, Hopkinton, MA). The MSCs were suspended in Plasma-Lyte in a total volume of 60 ml. Administration proceeded over 30 min for each time point, taking place at 6, 24, and 48 h PI. Animals received a dose of MSCs sufficient to contain approximately 5.66 × 10^7^ platelets/kg as described previously (40).

For extracorporeal life support (ECLS) treatment, either the Hemolung or the Novalung ECLS device was applied based on the study group. The systems were primed with 0.9% normal saline and received an initial bolus of 5,000 units of unfractionated heparin plus a continuous infusion of heparin to maintain an activated clotting time (ACT) above 50 s. The ventilator settings of animals on ECLS were reduced as much as possible to reduce peak while maintaining normocarbia.

### 2.3 Vital signs and sample collection

Vital signs including heart rate and arterial blood pressure were monitored via a high-pressure monitoring line (Smith Medical ASD Inc., Dublin, OH) connected to the femoral artery catheter and recorded and stored using proprietary data acquisition software [Integrated Data Exchange and Archival (IDEA) system]. Arterial partial pressure of oxygen (pO_2_), partial pressure of carbon dioxide (pCO_2_), and arterial blood gas analysis was performed at the bedside using an iSTAT 300-G blood analyzer (Abbott Point of Care Inc., Princeton, NJ; VetScan CG4+ and CG8+ cartridges, Abaxis Inc., Union City, CA).

Serum samples were drawn at BL, PI, and 1, 2, 3, 6, 12, 24, 48, and 72 h PI, when applicable. After drawing, the serum samples were processed, aliquoted, and stored at −80°C and analyzed thereafter.

Tissue samples were collected at necropsy and subsequently processed for use in histological evaluation and immunohistochemistry assays.

### 2.4 Measurement of inflammatory mediators by enzyme-linked immunosorbent assay

Quantitative analysis of inflammatory mediators HMGB1, SDC-1, and C3a in serum samples was performed using commercial enzyme-linked immunosorbent assay (ELISA) kits according to the manufacturer’s recommendations. The ELISA kits of HMGB1 (IBL International, Cat# 30164033), SDC-1 (Cloud-Clone Corporation, Cat# SEB966Po), and C3a (MyBioSource, Cat# MBS2509360) were used for measuring HMGB1, SDC-1, and C3a, respectively.

### 2.5 Immunohistochemistry assay

Lung tissue was processed for immunohistochemistry (IHC) staining as described previously (41). Tissues were fixed with 10% neutral buffered formalin, and subsequent antigen retrieval was performed to restore antigenicity using a heat-induced epitope retrieval (HIER) method. After permeabilization and blocking, the slides were incubated with primary antibodies, including rabbit polyclonal to HMGB1 [IgG, polyclonal, cat#ab18256, diluted in 1% goat serum with phosphate-buffered saline with Tween (PBST) (Triton X-100, 0.1%), Abcam, Cambridge, MA] and mouse monoclonal to TLR4 [IgG2b, monoclonal, cat#ab22048, diluted in 1% goat serum with PBST (Triton X-100, 0.01%), Abcam, Cambridge, MA]. Following extensive washing, sections were incubated with secondary antibodies labeled with Alexa Fluor 488 (Green) or 594 (Red) (Abcam, Cambridge, MA) for 1 h at room temperature (RT). After washing, sections were mounted with ProLong Gold Antifade solution containing 4′,6-diamidino-2-phenylindole (DAPI) (Invitrogen, Carlsbad, CA) for staining the nuclear DNA. Then, the mounted tissue was visualized at 200× magnification using a Zeiss AX10 fluorescence microscope (Zeiss AX10).

### 2.6 Histopathological evaluation

Following euthanasia or immediately following death if prior to the 72-h timepoint, lung tissue samples were fixed in 10% normal buffered formalin (NBF), embedded into paraffin, sectioned at 4µm, and stained with hematoxylin and eosin (H&E). Histologic images were recorded with a 10× objective under a slide scanner (Axio Scan.Z1 v1.0, Zeiss, Germany).

### 2.7 Statistical analysis

Vital signs and blood gas data were presented as mean and standard deviation (SD), and a Mann–Whitney U test followed by a Dunn’s *post-hoc* test was applied for the statistical analyses. Longitudinal data were presented as mean and standard error of the mean (SEM). Since some data points were absent in the longitudinal data, a mixed-effect model for repeated measures with random intercept and random slope or a Friedman test followed by a Dunnett’s or Dunn’s *post-hoc* test where appropriate was used to examine within-group-specific differences in defined HMGB1, SDC-1, and C3a biomarkers throughout the observation period after injury, and each time point by the groups without ARDS and with ARDS as fixed effects. Receiver operating characteristic (ROC) curves were plotted for HMGB1, SDC-1, C3a, mean arterial pressure (MAP), PCO_2_, and oxygen saturation (SPO_2_) for predicting likelihood of ARDS or mortality for all conditions at 6 h PI. The optimal cutoff values with the Youden index and the areas under the ROC curves (AUROC) were calculated. Sensitivity and specificity using the optimal cutoff values for predicting outcomes were also performed. Logistic regression analysis was used for calculating odds ratios (ORs) with 95% confidence intervals (95% CI) for clinical outcomes of ARDS and mortality. Statistical significance was determined with a two-sided p < 0.05. Statistical analyses were performed using GraphPad Prism 9.3.1 (GraphPad Software, San Diego, CA).

## 3 Results

### 3.1 ARDS, survival, and blood gas monitoring

**Figure 1:**
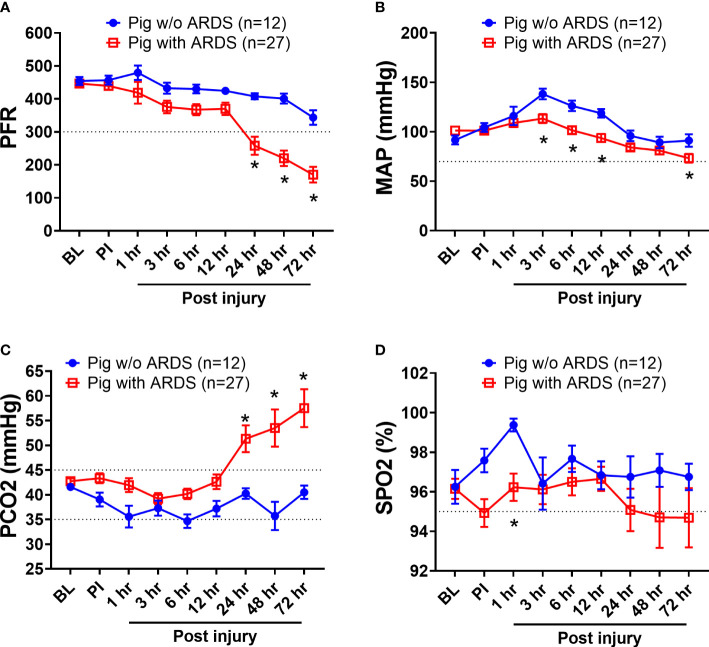
The clinical variables of animals that underwent smoke inhalation and burn injury. Data are presented as mean ± SEM and were statistically analyzed using the mixed-effects model for repeated measures. *p<0.05, the value of individual time point of swine with ARDS (n=27) vs. swine w/o ARDS (n=12). PFR **(A)**; MAP **(B)**, PCO2 **(C)**, SPO2 **(D)**, the partial pressure of carbon dioxide. The normal physiological ranges for each individual parameters are indicated by dotted lines.

Among the 39 swine monitored in this study, 27 (69.2%) animals developed ARDS, as determined by PFR, with a median time of 23 h (interquartile ranges, 9–43) PI. ARDS did not develop in the other 12 swine (30.8%) over the entire 72-h period in the ICU (**Figure 1A**). There were 10 (26%) swine that died, and the remaining 29 (74%) survived the follow-on observation period. The PFR values showed a statistical difference (p < 0.01) at 24 h PI through the remaining time points between swine with ARDS compared with those without ARDS (**Figure 1A**). MAP significantly dropped in swine with ARDS compared with those without ARDS from 3 to 12 h PI, and at the final time point. All the swine retained a MAP above the normal range (70 mmHg) (**Figure 1B**). PCO_2_ was significantly (p < 0.01) higher in swine with ARDS at 24 h PI compared with swine without ARDS; however, before 24 h PI, the PCO_2_ was well within the normal physiological range (35–45 mmHg) (**Figure 1C**). The SPO_2_ showed transient drops in the ARDS group at 1 h PI but recovered to above normal range (95%) thereafter. SPO_2_ appeared to decrease after 24 h PI, but no statistical difference was observed (**Figure 1D**). Interestingly, other clinical variables of PO_2_, base excess/base deficit (BE/BD), lactate, glucose, hematocrit (Hct), and hemoglobin (Hgb), all failed to show any statistical difference during the first 48 h PI (**Table 1**); nevertheless, both BE/BD and lactate showed statistical differences at 72 h PI (**Table 1**).

**Table 1:** The vital signs and blood gas data of animals that underwent smoke inhalation and burn injury.

Parameters	Groups	BL	PI	1 hr	3 hr	6 hr	12 hr	24 hr	48 hr	72 hr PI	Reference range^#^
**PO_2_ (mmHg)**	w/o ARDS (n=12)	95.00 ± 8.97	118.83 ± 80.35	95.67 ± 12.32	106.75 ± 49.94	93.00 ± 9.15	94.08 ± 14.45	94.92 ± 19.50	108.50 ± 46.21	113.17 ± 43.21	75-100 (42)
	with ARDS (n=27)	89.67 ± 15.72	121.88 ± 105.88	92.16 ± 30.62	94.60 ± 26.80	90.48 ± 24.15	101.15 ± 22.55	93.64 ± 22.01	102.59 ± 54.74	111.29 ± 91.47	
**BE/BD (mmol/L)**	w/o ARDS (n=12)	7.35 ± 4.83	6.69 ± 4.51	4.83 ± 6.67	4.50 ± 2.61	4.13 ± 2.33	2.48 ± 1.75	4.18 ± 2.99	5.31 ± 2.26	5.58 ± 2.94	-4 to +2 (42)
	with ARDS (n=27)	6.82 ± 3.38	7.20 ± 4.80	5.25 ± 3.28	5.03 ± 3.56	3.98 ± 3.23	2.01 ± 3.46	2.77 ± 4.79	7.78 ± 4.19	10.00 ± 8.27*	
**Lactate (mmol/L)**	w/o ARDS (n=12)	3.52 ± 3.62	4.17 ± 3.70	4.39 ± 4.12	2.34 ± 2.84	1.10 ± 0.59	0.75 ± 0.26	0.77 ± 0.63	0.61 ± 0.32	0.56 ± 0.23	0.5-5.5 (43)
	with ARDS (n=27)	2.03 ± 1.30	2.95 ± 1.82	2.35 ± 2.01	1.80 ± 1.05	1.40 ± 0.90	1.12 ± 1.32	1.62 ± 1.90	1.01 ± 0.82	1.75 ± 2.70*	
**Glucose (mg/dL)**	w/o ARDS (n=12)	124.75 ± 22.34	192.83 ± 56.27	108.25 ± 33.39	96.00 ± 31.35	95.17 ± 15.03	95.50 ± 11.90	87.92 ± 38.04	86.64 ± 27.68	72.17 ± 11.82	52–153.88 (44)
	with ARDS (n=27)	138.73 ± 31.16	214.37 ± 58.32	121.29 ± 24.83	93.60 ± 14.98	98.57 ± 21.93	88.46 ± 19.12	98.68 ± 37.06	81.88 ± 36.82	62.38 ± 30.46	
**Hct (%)**	w/o ARDS (n=12)	29.58 ± 3.53	34.91 ± 2.96	30.80 ± 3.83	27.38 ± 3.45	27.04 ± 3.65	26.38 ± 2.45	22.88 ± 3.84	18.78 ± 3.83	16.58 ± 2.71	25.38–38.77 (44)
	with ARDS (n=27)	30.75 ± 3.60	34.54 ± 4.62	34.51 ± 6.13	28.48 ± 5.54	30.29 ± 5.36	28.09 ± 5.39	25.33 ± 5.03	20.70 ± 4.07	18.75 ± 4.09	
**Hgb (mmol/L)**	w/o ARDS (n=12)	8.98 ± 1.71	10.40 ± 1.43	10.90 ± 1.52	9.35 ± 1.14	7.68 ± 1.37	7.25 ± 1.26	7.11 ± 1.01	5.86 ± 0.95	5.13 ± 0.99	8.20–11.7 (44)
	with ARDS (n=27)	9.53 ± 2.31	10.28 ± 1.38	10.97 ± 1.17	9.60 ± 1.50	8.52 ± 1.52	7.75 ± 1.86	7.22 ± 1.42	6.40 ± 1.53	5.47 ± 1.04	

BL, baseline; PI, post-injury; BE/BD, base excess/base deficit; Hct, hematocrit; Hgb, hemoglobin. Data were presented as mean ± SD, and statistical analyses were performed by the Mann–Whitney U test. *p<0.05, w/o ARDS vs. with ARDS. Significant differences are indicated by boldface type. ^#^Reference ranges for PO_2_ and BE/BD are based on data from humans, and the reference ranges for lactate, glucose, Hgb, and Hct are from swine studies.

### 3.2 HMGB1 is significantly increased in injured animals that underwent smoke inhalation and burn injury

**Figure 2:**
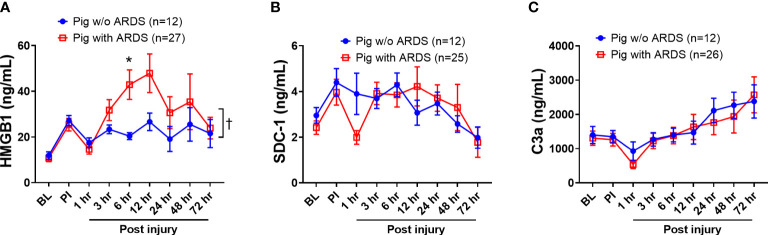
The dynamic changes of inflammatory mediators in the blood of animals with ARDS development and without (w/o) ARDS after smoke inhalation and burn injury. A pooled sample set from three swine studies (n=39) underwent smoke inhalation and 40% total body surface area (TBSA) burn, followed by mock treatment, or therapeutic treatments in ICU care for up to 72 h PI or unless early death. The serum samples were taken at BL, PI, and 1, 3, 6, 12, 24, 48, and 72 h PI, and the inflammatory mediators of HMGB1 (**A**, pig with ARDS n=27; pig w/o ARDS n=12), SDC-1 (**B**, pig with ARDS n=25; pig w/o ARDS n=12), and C3a (**C**, pig with ARDS n=26; pig w/o ARDS n=12) in samples were measured by individual ELISA kits respectively, and the data were presented as mean ± SEM. Statistical analyses were performed by the linear mixed-effect model for repeated measures, and for the least square means of individual group comparisons. *p<0.05, the value of individual time point of swine with ARDS vs. w/o ARDS; and † p<0.05, for the least square means between the groups.

HMGB1, SDC-1, and C3a, which are key biomarkers representative of three respective pathways—damage-associated molecular patterns (DAMPs), endotheliopathy, and complementopathy (45, 46)—were evaluated in the blood serum. At 6 h PI, HMGB1 levels in swine that developed ARDS significantly increased (p < 0.05) compared with those swine that did not develop ARDS (**Figure 2A**); overall, serum HMGB1 significantly increased in swine with ARDS compared with those swine that did not develop ARDS (**Figure 2A**). The SDC-1 and C3a levels in serum among those swine with ARDS and those swine without ARDS were not statistically different (**Figures 2B, C**), although the observable C3a levels were increased in both groups (**Figure 2C**).

### 3.3 HMGB1 is correlated with mortality and incidence of ARDS

**Figure 3:**
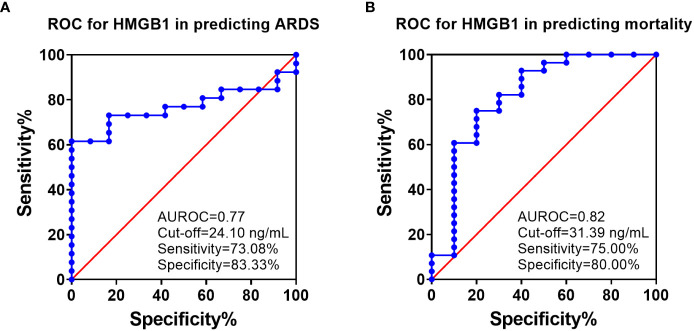
The receiver operating characteristic (ROC) analysis of HMGB1 with ARDS development and mortality. ROC curve plotted for studying of the levels of HMGB1 in the blood samples (6 h PI) in predicting of ARDS development **(A)** and mortality **(B)**. The area under the ROC (AUROC), the optimal cutoff value with Youden index, sensitivity, and specificity for each prediction are listed in the right-side panel of each graph.

ROC analysis showed that HMGB1 levels at 6 h PI is significantly associated with ARDS and mortality in swine exposed to SI and burn injury (**Figure 3**). For ARDS prediction, the area under the ROC curve was 0.77, and the optimal cutoff value with a Youden index of HMGB1 predicting ARDS was 24.10 ng/ml. The sensitivity and specificity using the optimal cutoff value for predicting the outcome were 73.08% and 83.33%, respectively (**Figure 3A**). For mortality prediction, the area under the ROC curve was 0.82 and the optimal cutoff value was 31.39 ng/ml. The sensitivity and specificity for predicting mortality were determined at 75.00% and 80.00%, respectively (**Figure 3B**). Interestingly, the ROC analysis also revealed that MAP and PCO_2_ are correlated with ARDS and mortality at 6 h PI; however, their cutoff values for prediction are all within normal physiological ranges: The MAP cutoffs were 113 and 104.5 mmHg for predicting ARDS and mortality (**Table 2**), and the PCO_2_ cutoffs were 36.8 and 37.45 mmHg for predicting ARDS and mortality, respectively (**Table 2**).

**Table 2:** ROC analysis of risk factors associated with ARDS and mortality at 6 hours PI.

ROC	AUROC	*p-*value	Cut-off	Sensitivity	Specificity
**SDC-1 with ARDS**	0.6	0.35	n/a	n/a	n/a
**C3a with ARDS**	0.57	0.47	n/a	n/a	n/a
**MAP with ARDS**	0.84	<0.01	113 mmHg	81.48	83.33
**PCO2 with ARDS**	0.74	0.01	36.8	62.96	66.67
**SpO2 with ARDS**	0.56	0.52	n/a	n/a	n/a
**SDC-1 with mortality**	0.54	0.74	n/a	n/a	n/a
**C3a with mortality**	0.66	0.14	n/a	n/a	n/a
**MAP with mortality**	0.82	<0.01	104. 5 mmHg	75.86	80
**PCO2 with mortality**	0.82	<0.01	37.45	68.97	80
**SpO2 with mortality**	0.53	0.75	n/a	n/a	n/a

ROC, receiver operating characteristic; AUROC, area under the receiver operating characteristic; SDC-1, syndecan-1; MAP, mean arterial pressure; PCO2, The partial pressure of carbon dioxide; n/a, not applicable.

Logistic regression analyses show that HMGB1 levels are significantly associated with clinical outcomes of ARDS and mortality. At 6 h PI, animals with HMGB1 levels ≥24.10 ng/ml were associated with a 13-fold higher risk of ARDS [OR:13.57 (2.76–104.3), p=0.01] and animals with HMGB1 levels ≥31.39 ng/ml were associated with a 12-fold higher risk of mortality [OR: 12.00 (2.36–93.47), p = 0.01] (**Table 3**).

**Table 3:** Factors associated with odds of ARDS and mortality.

Univariate	ARDS		Mortality	
	OR (95% CI)	*p*-value	OR (95% CI)	*p*-value
**HMGB1**	1.09 (1.03-1.22)	0.01	1.05 (1.02-1.10)	<0.01
**HMGB1 ≥24.10 vs. <24.10**	13.57 (2.76-104.3)	0.01	n/a	n/a
**HMGB1 ≥31.39 vs. <31.39**	n/a	n/a	12.00 (2.36-93.47)	0.01
**SDC-1**	0.91 (0.66-1.27)	0.35	1.21 (0.87-1.72)	0.74
**C3a**	1.00 (1.00-1.00)	0.47	1.00 (1.00-1.00)	0.15
**PFR**	1.00 (0.98-1.00)	0.01	0.97 (0.97-1.00)	0.01
**MAP**	0.93 (0.88-0.97)	<0.01	0.94 (0.88-0.98)	<0.01
**PCO_2_**	1.26 (1.07-1.58)	0.02	1.32 (1.12-1.66)	<0.01
**SPO_2_**	1.01 (0.97-1.15)	0.52	0.97 (0.79-1.03)	0.75

ARDS, acute respiratory distress syndrome; HMGB1, high-mobility group box 1; PFR, PO_2_/FiO_2_ ratio; MAP, mean arterial pressure; PCO_2_, the partial pressure of carbon dioxide; n/a, not applicable.

### 3.4 MSC treatment significantly reduced HMGB1 levels in circulation

**Figure 4:**
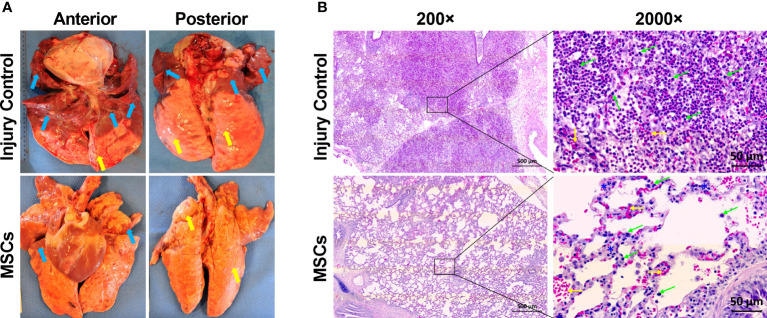
The representative macroscopic and microscopic images of the lung appearances in injured control and MSC-treated swine. The swine underwent smoke inhalation and burn injury, followed by mock treatment (saline), or autologous MSC treatment and in the ICU care for up to 72 h PI or unless early death. **(A)** The macroscopic views of lung tissues were taken at necropsy. Edema (yellow arrow) and large amounts of necrotic tissues or hematoma (blue arrows) were presented in the lung of injured control swine, but the MSC-treated swine was shown of much less of edema (yellow arrows) and necrotic damages (blue arrows) in the lung. **(B)** The histological images were stained with hematoxylin (H) and eosin (E). For the injured control swine, extreme cellular inflammatory infiltrates, with majority of macrophages and neutrophils (green arrows), were presented in the pulmonary tissue. Also, alveolar hemorrhage (yellow arrows) was presented in the lung tissue. For the MSC-treated swine, much less of inflammatory infiltrates (green arrows) with septal thickening (blue stars) and alveolar hemorrhage (yellow arrows) were presented in the tissue. The magnification is 200× for the left panels and 2,000× for the right panels.

Previously, we found that MSC treatment reduced ARDS in swine with smoke inhalation and burn injury, but the molecular mechanisms for this reduction remain unclear (40). In the current study, we found that MSC treatment reduced tissue edema and decreased the amount of necrotic damage and/or hematomata in the lung (**Figure 4A**). The MSC treatment also reduced inflammatory cell infiltration. Most of these infiltrates were presented as macrophages and neutrophils (**Figure 4B**) in the injured animal, which were largely reduced after MSC treatment.

**Figure 5:**
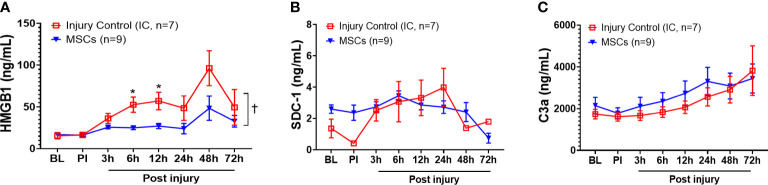
Efficacy of inflammatory mediator responses in the blood of injured control and MSC-treated swine. The swine were sustained to smoke inhalation and burn injury then treated with saline (injury control, n=7), or autologous MSC treatment (MSC, n=9), and observation for up to 72 h PI. The blood samples were taken at BL, PI, and 3, 6, 12, 24, 48, and 72 h PI, and the inflammatory mediators of HMGB1 **(A)**, SDC-1 **(B)**, and C3a **(C)** were measured by individual ELISA kits respectively, and the data were presented as mean ± SEM. Statistical analyses were performed by the linear mixed-effect model for repeated measures, and for the least square means of individual group comparisons. *p<0.05, the value of individual time point of IC vs. MSC; and †p<0.05, for the least square means between the groups.

Among the inflammatory mediators tested, HMGB1, SDC-1, and C3a results showed that MSC treatment significantly reduced overall serum HMGB1 in the MSC group compared with the injury control group (**Figure 5A**) but did not reduce SDC-1 and C3a levels (**Figures 5B, C**). Specifically, MSC treatment significantly reduced serum HMGB1 concentrations at 6 and 12 h PI (**Figure 5A**).

### 3.5 MSC treatment significantly reduced the HMGB-TLR signal pathway activation in the lung

**Figure 6:**
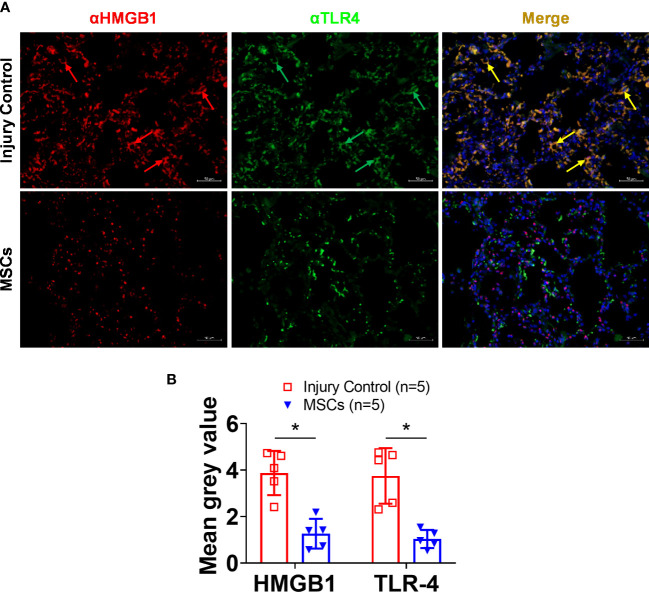
HMGB1 and TLR-4 expressions in the lung tissue of injured control and MSC-treated swine. The swine were exposed to smoke inhalation and burn injury, then treated with saline (injury control), or autologous MSC treatment (MSC), and observation for up to 72 h PI. **(A)** Lung tissue samples were obtained during necropsy and proceeded for immunohistochemistry (IHC) staining, the primary antibodies of anti-HMGB1 (rabbit anti-HMGB1), and anti-TLR4 (mouse anti-TLR4), followed by visualization with a goat anti-rabbit IgG conjugated with Cy3 (red) and goat anti-mouse IgG conjugated with cy2 (green), respectively. DNA was stained with 4′,6-diamidino-2-phenylindole (DAPI) (blue). Scale bar = 50 μm. Note that the co-localized staining (yellow arrows) of HMGB1 and TLR4 was observed in the injury control but not in the MSC treatment group. **(B)** The mean immunofluorescence signal intensity in the IHC images was measured by ImageJ software and presented as mean± SEM. n=5 swine per experimental group was analyzed. Statistical analysis was performed using the Mann–Whitney U test, *p<0.05.

IHC analysis of HMGB1 and TLR4 showed strong activation in the lung after smoke inhalation and burn injury (**Figure 5**), and subsequent MSC treatment led to significantly reduced expression of both HMGB1 and TLR4. Interestingly, a co-localization of HMGB1 and TLR4 was observed in the injured animals, and subsequent MSC treatment resulted in reduced HMGB1, TLR4, and HMGB1-TLR4 complex co-localization in the lung (**Figure 6**).

## 4 Discussion

Previous studies suggest that DAMPs, endothelial, and complement pathways are associated with organ failure and tissue damage (46–48). Three potential biomarkers in those categories were tested in our study (HMGB1, SDC-1, and Ca3), but only one showed a correlation with development of ARDS in our model. The serum concentration of HMGB1 was significantly increased in swine that developed ARDS compared with swine that did not develop ARDS. ROC analyses showed a strong correlation between HMGB1 and ARDS progression as well as mortality with high specificity and sensitivity. Importantly, this correlation is evident as early as 6 h PI versus the clinical standards approach by PFR where the median time to diagnosis was 23 h PI. Clinically, ARDS diagnoses can take anywhere between 24 and 48 h to confirm because of the requirement for symptoms to present in conjunction with a number of clinical criteria that must be met. The Berlin criteria cutoff is 1 week. This is obviously problematic for medical providers as ARDS can progress within hours and lead to mortality at any time during that period. These limitations highlight the need for a diagnostic test that can provide vital insight to a patient’s risk of developing a severe respiratory disease.

Of the three research studies examined, one was designed to test the effect of MSC intervention on the incidence of ARDS using burn and smoke inhalation injury. While the MSC therapeutic potential could not be conclusively determined in this study, the correlation between HMGB1 levels and ARDS development was strongly evident. Notably, the concentration of HMGB1 in the MSC treatment group was significantly lower than that of the untreated group. Likewise, our results showed high expression and co-localization of HMGB1-TLR4 in the lungs of injured animals, whereas the MSC treatment group showed significantly reduced HMGB1 and TLR4 expression as well as decreased co-localization in the lung. Additionally, when results from all three studies are pooled together as in **Figures 1**, **2**, these originally independent studies collectively indicate a strong relationship between the presence of HMGB1 in circulation (and further at the tissue level for MSC treatment) in the development of ARDS.

Many pharmacological studies in small animal models have demonstrated promise, but those treatments ultimately failed when moved to clinical trials (49). However, compared with small animals, large animals such as swine are ideal for screening and testing therapeutic approaches thoroughly before moving to clinical trials with a high risk of failure. Swine anatomical, physiological, immunological, and disease progressions share translative similarities to humans (50, 51). Our facility has a well-established platform for managing large animal care under ICU conditions, utilizing the same equipment present in our adjacently located Intensive Critical Care Unit and Level 1 Trauma center at Brooke Army Medical Center, mimicking almost all aspects of clinical care for human trauma patients (37, 40, 52).

Typically, HMGB1 is known as a nuclear DNA binding protein, but recently it has emerged as an important DAMP molecule that moderates inflammation and immune response (24), and it can be passively released from damaged cells or actively secreted from activated immune cells (53). Elevated levels of HMGB1 are associated with many inflammatory-mediated diseases and organ injuries such as lung injury, myocardial infarction, and rheumatoid arthritis (54–56). Our results identified HMGB1 as a viable predictor of ARDS and mortality after traumatic injury, as indicated by inflammatory response and cellular damage. In this pooled study, 39 animals were included, and all animals underwent smoke inhalation and burn injury. Afterward, the animals received different treatments (nine swine with autologous MSCs, seven swine with Novalung, six swine with Hemolung, eight swine with saline, and nine swine without any fluid resuscitation). The diversity of treatments strengthens the results reliability and clinical relevance, as there is also a high diversity of ARDS patients in clinical settings (57, 58). This result is consistent with previous work proposing other biomarkers as predictors of ARDS, including IL-6 (59), IL-8 (60), RAGE (61), C-reactive protein (62), and surfactant protein D (SPD) (63). However, the use of these biomarkers still faces barriers toward clinical use. Due to the heterogeneity of the onset of ARDS, the utility of some biomarkers may be limited, such as SPD which may only correlate with ARDS caused by direct lung injury, but not indirect lung injury (64). The diagnostic value of other potential markers has yet to be definitively evaluated and remains unverified (65). What makes HMGB1 appealing are its unique characteristics that separate it from other inflammatory biomarkers. First, HMGB1 is an early release inflammatory marker following cell injury, as reported in previous works by our group (48) and others (66). Once HMGB1 is released, it can trigger downstream cytokine release (67), endothelial damage (68), and oxidant release (69). Second, HMGB1 acts through a positive feedback mechanism as damaged cells further induce additional HMGB1 release (70). In the current study, HMGB1 levels initially declined 1-h PI but subsequently increased beyond BL 6 h PI (**Figure 1A**). Third, HMGB1 elevation has been reported in many types of injuries, such as severe trauma (48), hemorrhage (48), lipopolysaccharide (LPS) stimulation, and sepsis (71). Fourth, neutralization of HMGB1 in bronchoalveolar lavage (BAL) fluid in ARDS patients improved efferocytosis and neutrophil extracellular trap (NET) clearance (72), all of which are potential precursors that can precipitate ARDS progression. These distinctive characteristics may contribute to the correlation between HMGB1, the development of ARDS, and survival.

In addition to HMGB1 changes, there was a statistically significant difference to MAP and PCO_2_ at 6 h PI to both the development of ARDS and mortality as evaluated by ROC analysis (**Table 2**). Cutoff values of 113 and 104.5 mmHg for MAP and 36.8 and 37.45 mmHg for PCO_2_ correlated with ARDS development and mortality, respectively. While interesting, taken alone these data have limited clinical use, as the cutoff values of both MAP and PCO_2_ are within normal ranges and blood pressure is particularly labile (73). Although the PCO_2_ data increase in swine with ARDS compared with swine without ARDS (**Figure 1C**), this occurs relatively late PI (24 h after injury), and the mechanism of reduced gas exchange might be due to impaired alveolar fluid clearance as previously reported (21). Additionally, when considering the disparate conditions and diseases that may contribute to a change in MAP and PCO_2_ in the clinical setting (74, 75), it becomes evident that broad metrics like these cannot contribute meaningfully to a differential diagnoses of a single disease.

HMGB1 has been previously proposed as a biomarker for differing traumatic injuries. Wang et al. reported that plasma HMGB1 was significantly higher in traumatic brain injury (TBI) patients compared with healthy controls, and plasma HGMB1 levels >10.8 ng/ml therein accurately predicted a 1-year unfavorable outcome of patients (76). Others also found that HMGB1 levels in cerebrospinal fluid (CSF) associated with poor clinical outcomes in pediatric TBI patients (77). HMGB1 could also serve as a diagnostic marker for severe blunt chest trauma (78) and acute appendicitis (79) and is associated with poor clinical outcomes in pediatric patients with acute traumatic coagulopathy (ATC) (80). While we propose that HMGB1 could serve as a prognostic marker for ARDS in a military related trauma model, potentially adding valuable information for early diagnosis of ARDS, it should be noted that due to the complexity of the pathogenesis of ARDS, many risk factors other than inflammatory response are currently under investigation for their relationship with ARDS development, including circular RNA (81), microRNA (82), factors related to mitochondrial dysfunction (83), metabolomics (84), or even a combination of two biomarkers (36) or a panel of biomarkers (85).

HMGB1 is a diverse biomolecule that acts on the body through signaling functions as well as mechanistically in homologous or heterogenous enzyme complexes. In our study, we examined the presence of HMGB1 in circulation and complexed with TLR4, but not in any other state. Our large animal model focused on smoke and burn-related causality, but omitted hemorrhage, and therefore identification of any potential contributors to ARDS progression by associated traumatic responses to severe bleeding injuries or exacerbation of injury due to improper resuscitation. Aside from DAMPs, endothelial dysfunction and complementopathy also contributed to trauma-induced organ failure (45, 46). We also noticed that SDC-1 was not correlated with ARDS development in our model (**Figure 1B**), which was initially unexpected. Although the presence of SDC-1 has previously been identified in other injury models, it should be noted that SDC-1 is evidently associated with vascular injury, such as severe hemorrhage (86). Consequently, SDC-1 has been used as a sensitive biomarker for patients with vascular damage (87), but it appears that this response does not translate to local tissue damage via smoke or burn injury of the lung or skin, respectively. A study using our traumatic hemorrhage swine model, which inherently incorporates the presence of vascular damage, will need to be assessed in future efforts to evaluate the predictive potential of SDC-1 in ARDS progression, as combat casualties will often suffer a traumatic hemorrhage in addition to other injuries such as smoke or burn exposure. Likewise, we did not identify an association between C3a and ARDS in our smoke and burn injury model (**Figure 1C**). As previously reported, large efforts have been made to associate complement activation in polytrauma, hemorrhage, and other injury types (47, 88), but research into complement activation in smoke inhalation and burn injury is limited. Our results suggest it is possible the type of injury inflicted in our model does not activate the classical or Lectin pathway that induces the C3a cascade (89, 90), which is associated with pathogen invasion but not traumatic injury. Furthermore, other cytokines and chemokines might also contribute to the phenotype of trauma-induced organ dysfunction (91), but further investigation is needed to determine precisely which of these biomolecules participate and to what extent.

Currently, ARDS constitutes a severe form of hypoxemia-induced respiratory failure with no existing FDA-approved pharmacological therapy for treatment. Treatment standards rely on supportive techniques that carry increasing risks such as VILI, hypervolemia, pulmonary edema, nephrotoxicity, and MOF the longer the treatment is in place but do not directly address the pathology of disease progression, increasing the likelihood for a drawn-out recovery process and poor patient outcomes (30–34). A major treatment challenge is due to the complexity of the pathophysiological mechanism and the heterogenous etiology of ARDS that elude discovery of optimal therapeutic targets for the disease (6, 58). MSCs are a heterogenous population of cells with multipotent differentiation that exhibit high potential for increasing angiogenesis, immunomodulation, and therapeutic functions (92). Recently, many *in vitro* and *in vivo* studies have shown that MSCs can modulate inflammatory pathways, improve organ function, and increase survivability through their paracrine effects (92). The data presented here show that MSC treatment led to significantly reduced HMGB1 expression, especially at 6- to 12-h PI time points. Although the efficacy of MSC treatment for improving survival still needs to be verified, our results imply that MSC treatment led to a reduced incidence of ARDS development. While eight out of eight non-treated swine developed ARDS, only four out of nine swine developed ARDS after MSC treatment. While further studies need to be performed, it could be likely that the MSC-induced attenuation of HMGB1 contributed to the reduced incidence of ARDS in this treatment group and that HMGB1 is a practical target for treatment or prevention of ARDS as demonstrated in this and previous studies (48, 93). It is interesting that HMGB1 presented in the cytosol of lung tissues (**Figure 6**) which may indicate active secretion of HMGB1 in response to smoke inhalation and burn injury. However, further examination is required to elucidate the cell type responsible for the HMGB1 intracellular expression, but these efforts are ongoing. It should also be noted that SDC-1 levels were unchanged after MSC treatment despite previously reported injury models showing reduced endothelial damage in MSC-treated groups; and the reasons why are still unclear (94).

While the results of this study are very promising, further evaluation will be required to determine HMGB1 and other biomarkers’ efficacy as clinical predictors of ARDS disease progression. This study served as a broad evaluation of potential biomarkers in multiple, differing scenarios based on the availability of samples. As such, tissue and blood samples, as well as vital signs, were obtained from three different studies carried out over the past 10 years all with objectives independent from our study aims. While the occurrence of a correlation between HMGB1 and ARDS progression exists in all scenarios, providing strong evidence that HMGB1 plays a vital role in this pathophysiology, our control over variables or elected time points was restricted. We were able to identify significance of HMGB1 levels and ARDS progression as early as 6 h, but the limited sample size at 3 h PI was not significant, even though clear differences were evident. Therefore, it will be prudent to design a research study specific to our research aim to verify our results in this study. Even though the studies were diverse in research objective and treatment method, they utilized the same smoke and burn model, which has limitations in the types of immune and traumatic injury responses activated. TBI, severe hemorrhage, or lung contusion are all common injury types a traumatic casualty may suffer, and incorporation of some or all of these injuries into our model would be required to develop a concise illustration of the pathophysiology of ARDS progression as it relates to HMGB1 or other biomarkers. Lastly, this study examined the co-localization of HMGB1 and TLR4. It will also be prudent in future studies to examine the relationship between several HMGB1 conjugates such as RAGE, TLR9, and complement to provide a more complete assessment of the contributions of HMGB1 toward activation of the immune response in traumatic injuries (95).

## 5 Conclusion

Using a swine model of smoke inhalation and burn injury, we identified HMGB1 as a potentially key biomarker for the early detection of ARDS. Upon traumatic injury, HMGB1 levels rise in circulation and in the tissues associated with injury. This systemic and local increase in HMGB1 expression and activity is strongly correlated and predictive for the development of ARDS in our large animal model. Importantly, this correlation is evident as early as 6 h PI versus clinical standards of diagnosis by PFR where the median time to diagnoses is 23 h PI and, thus, of significant clinical value as an early indicator of a severe respiratory disease. Observed differences were present at 3 h PI; however, the limited available sample size did not meet the threshold for statistical significance to correlate with ARDS development. Nonetheless, apparent changes are present, and it will be prudent to further investigate the correlation between HMGB1 and ARDS at earlier time points.

Our large animal model is more realistic compared with small animal studies and can produce translational results prior to advancing to clinical trials. The results of this study suggest that HMGB1 could potentially be a prognostic predictor of ARDS, especially in trauma-induced ARDS scenarios. Future studies will expand upon our model and scope to include more comprehensive disease progression and contributing pathophysiologic factors directed at HMGB1, its analogs, and other potential biomarkers to reliably anticipate the onset of ARDS in severe traumatic injuries.

## Data availability statement

The original contributions presented in the study are included in the article/supplementary material. Further inquiries can be directed to the corresponding authors.

## Ethics statement

The animal study was reviewed and approved by Institutional Animal Care and Use Committee (IACUC) of the US Army Institute of Surgical Research.

## Author contributions

Conceptualization: ZY and MY. Experimental and data analyses: ZY, TC, CT, RW, JKS, BJ, and MY. Project administration: ZY, MY, and JS. Supervision: MY and JS. Original draft preparation: ZY, TC, CT, RW, and MY. Reviewing and editing: ZY, TC, CT, RW, JKS, BJ, SD, MY, and JS. All authors read and approved the final manuscript.

## Author disclaimer

The views expressed in this article are those of the authors and do not reflect the official policy or position of the US Army Medical Department, Department of the Army, DoD, or the US Government. All research was conducted in compliance with the Animal Welfare Act, implementing Animal Welfare regulations, and the principles of the Guide for the Care and Use of Laboratory Animals. The Institutional Animal Care and Use Committee approved all research conducted in this study. The facility where this research was conducted is fully accredited by the AAALAC International.
